# Breast Cancer Incidence Among Patients Undergoing Chest Masculinization Surgery: A Scoping Review

**DOI:** 10.1245/s10434-025-18156-1

**Published:** 2025-08-28

**Authors:** Crystal Chu, Jennifer Goldman, Hyesong Joung, Sujeong Go, Randy Jones, John T. Stranix

**Affiliations:** 1 University of Virginia School of Nursing, Charlottesville, VA USA; 2 Department of Plastic Surgery, University of Virginia School of Medicine, Charlottesville, VA USA

**Keywords:** Breast cancer, Screening, Decision aid, DecisionKEYS, Shared decision making

## Abstract

**Background:**

The incidence of breast cancer (BC) cases among transgender males undergoing gender-affirming mastectomy (GAM) and the future BC risk for this population are not well established. This scoping review aimed to explore breast cancer incidence rates before and after GAM in the United States.

**Methods:**

Following the Arskey and O’Malley framework, the search was conducted in Embase and PubMed using keywords “gender-affirming surgery” and “breast cancer.” The initial search resulted in 405 articles, with 36 articles imported to Covidence for the screening and selection phase. The articles were limited to the United States alone and within the last 10 years.

**Results:**

For final inclusion, 13 articles were identified (11 observational/retrospective cohort studies and 2 case studies). Across all the studies, 42 cases of atypia, 6 cases of ductal carcinoma *in situ* (DCIS), 1 case of Paget’s disease, and 10 cases of invasive ductal carcinoma (IDC) were reported. Nine studies reviewed routine GAM surgical specimens (3869 cases), identifying 42 cases of atypia, 5 cases of DCIS, 1 case of Paget’s disease, and 3 cases of IDC. Seven invasive carcinomas and one DCIS case were detected pre-GAM during screening and involved concurrent treatment from breast and plastic surgery teams.

**Conclusions:**

Standardization and best-practice screening protocols, including breast imaging before GAM and pathology performed on specimens collected during GAM, are needed. A shared decision-making approach and clinical coordination, including breast and plastic surgery for patients who receive a breast cancer diagnosis while pursuing GAM, can help achieve oncologic and cosmetic goals.

Of the estimated 1.3 million adults in the United States who identify as transgender and gender non-binary (TGNB), reportedly 35.9% (480,000) are transgender men and 25.6% (341,800) are gender-nonconforming.^1^ Utilization of gender-affirming care is growing, with 25–35% of TGNB patients electing to undergo gender-affirming surgery (GAS)^2^ The most common initial procedure for transgender men and nonbinary individuals is gender-affirming mastectomy (GAM) or chest masculinization.

Because the goal of a GAM is to create a masculine chest contour, residual breast tissue remains at the time of mastectomy, and the incidence of future breast cancer in this population is not clear.^3,4^ It is well-established that of individuals assigned female at birth (AFAB), one will receive a diagnosis of breast cancer in her or his lifetime.^5^ Risk factors include age, family history, personal health history, and lifestyle factors. Current evidence, primarily based on cisgender AFAB individuals, indicates that the risk of breast cancer developing after GAM is low. Persons who identify as TGNB may possess unique risk factors such as exposure to hormonal therapy and variances in surgical tissue removal, which may affect their long-term breast cancer risk.

Despite the increasing number of TGNB individuals undergoing GAM, guidance on appropriate breast cancer screening after GAM is limited. Understanding breast cancer risk for this population is significant in the development of evidence-based screening recommendations. Therefore, this scoping review aimed to explore breast cancer incidence rates before and after GAM in the United States, and to synthesize key findings, including hormone therapy use and pathologic findings, to provide insights to inform future clinical guidelines.

## Methods

### Search Strategy

The methodology in this study was based on a modified version of the Arksey and O’Malley framework and the Preferred Reporting Items for Systematic Reviews and Meta-Analyses extension for Scoping Reviews (PRISMA-ScR) guidelines for conducting scoping reviews.^6,7^ A protocol was not prepared or registered before completion of this scoping review. The search was initiated by selecting specific keywords and controlled vocabulary. A health science librarian assisted in searching across PubMed and Embase.

The primary search centered around two main keywords, “gender-affirming surgery” and “breast cancer.” The initial search yielded 405 articles, which were imported into Zotero. After removal of 129 duplicate articles, the results were transferred to Covidence for the study selection and screening process.

### Inclusion and Exclusion Criteria

During the study selection phase, studies were included in the analysis if they were published in the English language within the last 10 years, investigated a population in the United States, and assessed breast cancer incidence among transgender individuals who underwent gender-affirming mastectomy. Studies were excluded if they were a review study including a systematic review and not available in a full text.

Two independent reviewers (H.J., S.G.) performed title/abstract review followed by full-text review. Screening disagreements were resolved by a third investigator (C.C.) with consensus discussions by R.J.

**Fig. 1:**
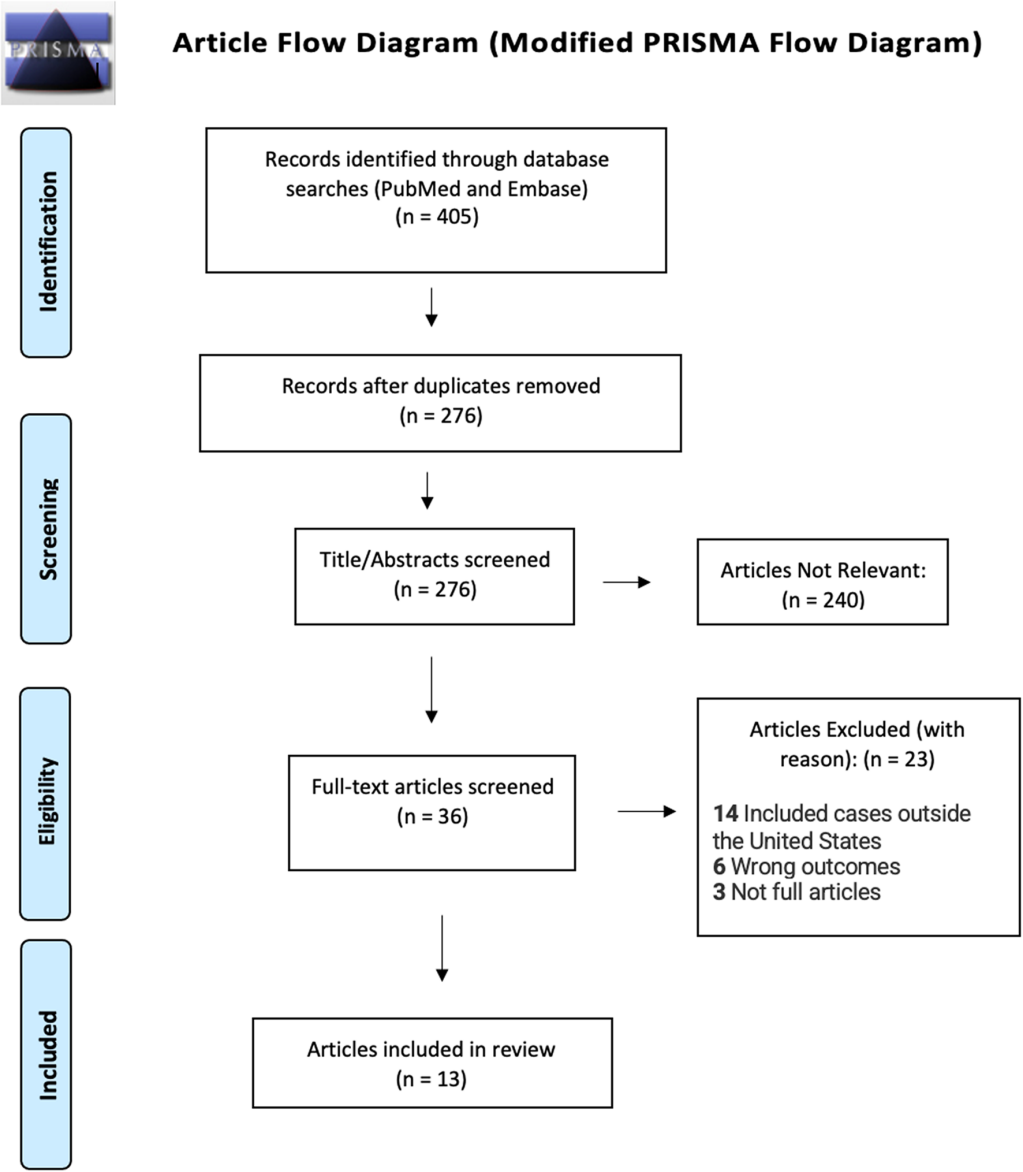
PRISMA-ScR flow diagram

After the title and abstract screening, 240 articles were deemed irrelevant and excluded, and 36 articles underwent a selection process by a review of the full text. Of these 36 articles, 23 were excluded based on criteria. The PRISMA-ScR flow diagram provides a detailed overview of the justifications for article exclusion (Fig. 1).

### Extracting and Synthesizing

Key data were systematically extracted from selected studies to evaluate the relationship between gender-affirming surgeries and breast cancer-related outcomes for transgender individuals. The extracted information included study characteristics such as author, year, aims, sample size, and design, as well as specific data on breast cancer outcomes and pathologic findings.

The process prioritized identifying the timing of breast cancer detection relative to GAM (e.g., cases identified before, after, or incidentally) and the types of breast cancer or precancerous conditions reported (e.g., atypical ductal hyperplasia [ADH], atypical lobular hyperplasia [ALH], ductal carcinoma in situ [DCI], invasive carcinoma). The study also included data on testosterone or androgen therapy as additional therapies that emerged as relevant factors. Additionally, data were recorded on preoperative risk assessments, pathologic sampling protocols, and the application of multidisciplinary approaches in managing oncologic and reconstructive outcomes. A summary chart of the assessment and key results of these studies is presented in Table 1.

**Table 1:** Study characteristics and results of published studies on histopathologic results for TGNB patients

Authors (year)	Study design	Sample	Results/findings	No. of pathologic results	Conclusions
Baker et al.^8^ (2021)	Retrospective cohort	447 Breast surgical specimens from transgender individuals undergoing gender-affirming chest-contouring surgery between 2013 and 2019	Subjects receiving testosterone therapy (TT) for ≥12 months: fewer cysts, fibroadenomas, PASH, columnar cell changes, papillomas, and mild inflammation Longer TT duration significantly associated increased lobular atrophy	Total number: 11 Atypia: 10 ADH: 7 ALH: 2 Both ADH and ALH: 1 DCIS: 1	Atypical lesions and DCIS were detected in 11 subjects on TT. TT does not significantly alter breast morphology. Culturally sensitive breast cancer screening was recommended for TM and GNC individuals.
Baker et al.^9^ (2019)	Retrospective cohort	340 Patients who underwent chest-contouring surgery between 2013 and 2018	1 Patient a diagnosis of ductal carcinoma *in situ* 3.5% had prior benign breast disease. 12 Breast cancer cases among 3698 TM, none with genetic risk characterization	Total number: 5 Atypia: 4 ADH: 2 ALH: 1 Both ADH and ALH: 1 DCIS: 1	Breast cancer risk factors (BMI, reproductive factors, family history, previous benign breast disease, alcohol consumption, tobacco smoking) are similar between TM and GNCIs regardless of TT.
Bhagat et al.^18^ (2022)	Case report	One 47-year-old African American transgender male found with a 3-cm malignant breast mass before planned GAM	Pathology-confirmed grade 2 IDC, ER-positive, PR weakly positive, HER2-negative, with BRCA2 positivity Multidisciplinary teams participated for surgery and adjuvant therapy. At 6 weeks post-operation, no complications, nipple graft failure, patient’s satisfaction	Total number: 1 IDC: 1	Surgical considerations for transgender patients differ from those for cisgender mastectomy cases. Screening guidelines for transgender patients are still undetermined. The impact of masculinizing hormone therapy on cancer incidence is unclear.
Boyd et al.^10^ (2022	Retrospective cohort	5 Transmasculine patients who underwent concurrent gender-affirming in the context of newly diagnosed breast cancer	All patients had sentinel lymph node biopsies. One required chemotherapy and radiation. No recurrence during follow-up (mean, 20.6 months) None had used TT	Total number: 5 IDC: 4 DCIS: 1	For oncologic safety and reconstructive success, a multidisciplinary approach is paramount. Standard oncologic follow-up should be done for all patients to assess for cancer recurrence regardless of gender-affirming procedures.
Bruce et al.^11^ (2022)	Retrospective cohort	318 TM patients undergoing chest reconstructive surgery	6.6% of patients had some increased risk of breast cancer. 5.3% had proliferative lesions. 0.6% had ADH. 0.6% had IDC. Two patients required chemotherapy and/or radiation.	Total number: 4 Atypia: 2 IDC: 2	Perform breast cancer risk assessment before surgery and use pathologic findings to guide postoperative cancer screening and follow-up. Due to residual breast tissue and possible effects of exogenous hormones, ensure regular clinical follow-up for all patients.
Fehl et al.^19^ (2019)	Case report	One case study of a 41-year-old transgender male diagnosed with IDC while receiving TT	Breast cancer risk in transgender men is reduced by the completion of bilateral mastectomy. Expense and complexity of name and gender identification changes lead to delays. Embarrassment in disclosing transgender status to health care providers Need for cultural competence among staff for safe gender identity disclosure	Total number: 1 Invasive carcinoma: 1	Tailored breast cancer screening guidelines are neeed for transgender individuals because their risks and presentation differ from those of cisgender patients.
Hernandez et al.^12^ (2020)	Retrospective cohort	211 Gender-affirming mastectomy cases	Significant findings were present in 6 of 211 GAM cases. By comparison, 19 of 273 reduction mammaplasty (RM) specimens yielded significant findings. In the gender-affirming group, 142 trans men underwent androgen therapy before surgery, 2 of whom had significant pathologic findings.	Total number: 6 Atypia: 6 LCIS & ALH: 1	Family history of breast cancer or the use of androgen therapy before surgery in gender-affirming individuals did not increase the risk of identifying significant breast lesions. The authors recommend submitting 4 tissue blocks per mastectomy for individuals undergoing gender-affirming breast surgery.
Istl et al.^13^ (2024)	Retrospective cohort	60 TGD patients with a diagnosis of cancer	5 Patients identified with breast cancer, 1 transgender male who had previously undergone GAM.	Total number: 1 IDC: 1 (BRCA positive)	16–20% of cancers diagnosed in the TGD population were cancers for which routine screening guidelines exist (breast, cervical, prostate, colon, and lung).
Kolbow et al.^14^ (2023)	Retrospective cohort	880 GAM patients 2010–2022	Of 880 patients included, 7 (0.8%) had a diagnosis of atypia, and none had a diagnosis of breast cancer. The median age at surgery for patients without and with atypia was 25.0 and 36.0 years, respectively.	Total number: 7 Atypia: 7 (Incidental cases at GAM: 7)	The risk of subsequent breast cancer after chest masculinization surgery is likely low for patients with or without a diagnosis of atypia at the time of surgery, but not all chest masculinization surgeries are equivalent.
McCaffrey et al.^15^ (2024)	Retrospective cohort	A total of 865 cases of patients who had GAM or breast reduction	High risk or malignant findings were noted in pathology results for 12 of 1730 breasts (0.7%). Patients younger than 25 years were 70% less likely to have any incidental finding on pathologic evaluation than older patients.	Total number: 6 Atypia: 4 DCIS: 1 Paget’s disease: 1 (Incidental cases at GAM: 6)	The overall incidence of cancer in TGD patients seeking top surgery is low. Patients undergoing GAHT should not be screened for breast cancer with increased frequency compared with cis-gender women
Parmeshwar et al.^3^ (2022)	Retrospective cohort	286 GAM patients	2 Patients (0.7%) with incidental breast cancer noted on their pathology specimen	Total number: 2 DCIS: 1 (DCIS w/ADH: 1) IDC: 1 (Incidental cases at GAM: 2)	Breast surgeons should counsel patients on the implications of residual breast tissue after GAM and the effect of hormone therapy on breast cancer risk.
Torous & Schnitt^16^ (2019)	Retrospective cohort	148 Patients that underwent GAM or breast reduction	Atypical ductal hyperplasia: 2% (3/148) *In-situ* carcinoma (LCIS, DCIS): 1% (1/148)	Total number: 6 Atypia: 5 ADH and ALH: 1 DCIS: 1	Epidemiologic and clinical studies have shown mixed results regarding the relationship between androgen administration and the risk for the development of breast cancer.
Wolters et al.^17^ (2023)	Retrospective cohort	374 Transgender bilateral mastectomy cases from 2017–2020 vs 127 cases of cisgender females undergoing elective breast reduction.	Breast specimens from trans men on AT vs cisgender women, showed a median higher gross percentage of fibrous tissue, apocrine metaplasia, calcifications, columnar cell change, and atypia	Total number: 4 Atypia: 4 (Incidental cases at GAM: 4)	Breast specimens from trans men, particularly those with a history of AT, or rare cases of atypia were not predicted by preoperative imaging or gross findings, supporting routine microscopic evaluation of these specimens

*TGNB* transgender and gender non-binary, *TT* testosterone therapy, *ADH* atypical ductal hyperplasia, *ALH* atypical lobular hyperplasia, DCIS ductal carcinoma in situ, *BMI* body mass index, *TM* transmasculine, *GAM* gender-affirming mastectomy, *IDC* invasive ductal carcinoma, *ER* estrogen receptor, *LCIS* lobular carcinoma in situ, *TGD* transgender and gender-diverse, *GAHT* gender-affirming hormone therapy, *AT* androgen therapy

## Results

Of the 13 studies included in this review, 11 were retrospective cohort studies,^3,8–17^ whereas 2 were case studies (Table 1).^18,19^ Medical records, including pathology reports from surgical specimens, were examined in all 11 retrospective studies conducted at a single academic institution. In two cases, a patient with plans to undergo GAM had invasive ductal carcinoma diagnosed before the surgery. Nine studies focused on reviewing surgical specimens or pathology data collected at the time of GAM,^3,8,9,12–17^ whereas four studies reported cases detected before GAM.^10,11,18,19^ In the cases identified before GAM, the patients presented with symptoms such as a palpable breast mass and subsequently underwent diagnostic procedures, including screening mammogram, as part of routine breast cancer screening.

Breast cancer diagnosed through pathology at the time of GAM was a rare finding. Among the 3869 cases included across all the studies, 6 cases were reported as DCIS,^3,8–10,16^ 10 cases were identified as invasive ductal carcinoma, ^3,10,11,13,18,19^ and 1 case was reported as Paget’s disease.^4^ Additionally, 42 cases of atypical lesions were documented, including ADH and ALH. The association between age and incidence of significant pathologic findings remains unknown because information regarding the age of the participants was inconsistently collected. Additionally, 5 of 11 retrospective cohort studies recorded additional information, including family history of breast cancer.^8,9,11,12,14,17^ However, the correlation between this information and the diagnosis of breast atypia or cancer remains unclear.

Testosterone therapy at the time of GAM was reported in 10 of the 13 studies,^3,8–10,12,14–17,19^ whereas 3 of the studies did not provide information on testosterone therapy.^11,13,18^ Among the 10 studies reporting testosterone use, 67–100% of the participants had received testosterone therapy, with varying durations of treatment. Notably, one study specified that none of the included patients had ever used testosterone therapy.^10^ Two studies reported that a longer duration of testosterone therapy was associated with increased lobular atrophy.^8,17^ However, the findings from four studies indicated that testosterone therapy did not correlate with an increased risk of clinically significant changes in breast morphology, such as higher frequencies of atypical lesions or carcinoma.^8,12,14,15^ Additionally, 4 of 10 studies did not assess or report the impact of testosterone therapy on breast tissue changes or the risk of significant pathologic findings.^5,9,16,19^

## Discussion

The primary aim of this scoping review was to evaluate the incidence of breast cancer among patients undergoing GAM. The main outcomes assessed were time of breast cancer finding (pre- or postoperative), pathology results, patient age and family history, and use of testosterone therapy. The primary findings suggested that incidental discovery of breast cancer at the time of GAM is uncommon, but not insignificant.

### The Importance of Routine Breast Screening

A few studies suggested that routine preoperative imaging tests for GAM cases might not be necessary.^12,14,17^ The low incidence of newly diagnosed breast cancer among patients undergoing GAM could support this. Nevertheless, 13.1% of AFAB individuals will have a diagnosis of breast cancer at some point during their lifetime, with percentages of new cases by age group increasing and peaking until the age of 65–74 years.^20^ The study of Lane et al.^21^ based on the Truven MarketScan Database found the average age of those undergoing GAM to be 28 years. Thus, most patients may not be at peak age of breast cancer risk when undergoing GAM. This is consistent with our findings showing such a low incidence of abnormal breast pathology.

It is important to recognize that, unlike mastectomy for breast cancer patients, GAM does not remove all breast tissue because a small amount of the tissue remains in place to contour the chest for aesthetic purposes.^22,23^ Thus, standard breast cancer screening should be encouraged by physicians once the patient reaches the recommended age. Those who have already reached the recommended age before GAM should be asked whether they have followed standard screening and, if not, they should complete the standard mammography before surgery. Proper intake at the time of the initial appointment should include relevant family medical history, previous benign breast disease, and reproductive factors related to increased risk.

Standardized recommendations for patients who are post-GAM for breast cancer screening are needed.^4^ Mixed guidelines recommend that patients can perform annual chest self-examination and that ultrasound or MRI may be appropriate depending on remaining tissue after surgery.^4^ Despite the sensitivity around breast cancer screening for TGNB individuals, health care providers should promote open communication to ensure high-quality, patient-centered care. Encouraging a proactive approach to breast cancer screening encompassing discussions of self-chest examinations and imaging recommendations could promote early detection and long-term health outcomes.

### Testosterone Therapy and Its Correlation with Breast Pathology

The results of testosterone therapy regarding abnormal breast pathology are mixed, with two studies reporting correlation with lobular atrophy and four studies indicating no correlation with significant changes in breast morphology. Although the majority of studies reported high rates of testosterone use, three studies did not, with one study explicitly noting that no patients received testosterone before surgery.

Testosterone is the cornerstone of therapy for transgender men to increase muscle mass and body hair and deepen the voice.^24^ With such a mixed review of results, further research is needed for systematic assessment of histopathologic changes in the context of testosterone therapy, particularly with standardized measures of exposure duration and dose. Additionally, chest-binding duration and number of hours per day as well as family history of breast cancer should be studied in parallel with evaluation for confounding factors.

Finally, no data are available on long-term effects of hormone therapy in breast pathology for TGNB patients. Given the increasing number of individuals undergoing GAM, further research is warranted to clarify the long-term implications of testosterone therapy for breast tissue morphology and oncologic risk.

### Strengths and Limitations

It is important to note the limitations of this study. First, the majority of the identified studies were retrospective cohort studies. The nature of this type of study limited our ability to assess the long-term incidence of breast cancer after GAM. The included studies lacked control groups, such as cisgender females who had undergone prophylactic or therapeutic mastectomy, for comparison regarding incidence of breast cancer after surgery. Variability in demographic reporting across studies (e.g., race, family history of breast cancer, history of benign breast disease) further complicated risk stratification and generalizability of findings. Further research must be conducted prospectively with a substantial follow-up period to properly evaluate the risk of breast cancer after GAM.

Additionally, the variability in testosterone therapy reporting across studies prevented us from establishing a definitive association between hormone therapy and abnormal breast pathologies. In future studies, it would be beneficial to incorporate not only testosterone therapy use but also other possible confounders, such as a history of chest-binding, in this patient population.

Our study also had several strengths. The large sample from multiple different centers allowed for adequate analysis of breast cancer incidence after GAM. Several factors involved in data collection, including breast pathology, testosterone use, and preoperative risk assessments, offered a multifaceted perspective. This included a variety of histopathologic findings, allowing for evaluation of pre-cancerous changes in TGNB patients. Finally, the use of a modified Arksey and O’Malley framework and PRISMA-ScR guidelines ensured a structured and transparent review process.

## Conclusion

It is imperative to continue standardized preoperative breast cancer screening for TGNB patients and to ensure that specimens collected during GAM undergo pathologic evaluation. Given the increasing number of individuals undergoing GAM. a collaborative approach among patients and surgeons is needed, as well as clinical coordination between specialties for the patients who receive a breast cancer diagnosis. Once patients have undergone GAM, health care providers should continue to educate them about future breast cancer screening needs including chest self-examinations and imaging. The integration of gender-affirming care into preventive health services is essential in providing equitable health care for TGNB individuals.
